# Oestrous females avoid mating in front of adult male bystanders in wild chacma baboons

**DOI:** 10.1098/rsos.181009

**Published:** 2019-01-16

**Authors:** Alice Baniel, Axelle Delaunay, Guy Cowlishaw, Elise Huchard

**Affiliations:** 1 Institute for Advanced Study in Toulouse, Toulouse, France; 2 ISEM, Univ Montpellier, CNRS, EPHE, IRD, Montpellier, France; 3 Institute of Zoology, Zoological Society of London, London, UK

**Keywords:** social influence, intersexual conflicts, intrasexual competition, chacma baboon

## Abstract

In social species, female mating strategies can be constrained by both male and female groupmates through sexual conflict and reproductive competition, respectively. This study tests if females adjust their sexual behaviour according to the presence of male and female bystanders in wild chacma baboons (*Papio ursinus*) and assesses their relative importance. Our results show that oestrous females initiate fewer copulations in the presence of adult male bystanders, irrespective of whether they are mate-guarded or not. This inhibitory effect probably reflects a response to indirect sexual coercion by males, whose close proximity may dissuade females to initiate copulations with rival males to avoid punishment and/or aggressive mating interference. By contrast, females initiate more matings with their mate-guard in the presence of higher-ranking female bystanders, which may reflect an attempt to secure bodyguard services from their mate when they feel threatened. These results emphasize the importance of intra- and intersexual conflicts in shaping female sexual behaviour in this promiscuous society.

## 1. Introduction

In social species, male and female groupmates frequently constrain the reproductive strategies of females. For example, dominant females often harass other females and interfere with their mating attempts, and monopolize resources that are necessary to breed, such as shelters, mates or offspring care [1]. Similarly, males may attempt to control female sexuality through the use of forced copulations, sexual harassment, intimidation and punishment [2–4].

In turn, females may evolve counter-strategies to alleviate the costs of social control, by inhibiting or hiding copulations in the presence of potentially aggressive bystanders [5,6]. In many promiscuous primates, copulations are conspicuous because females give loud vocalizations at the end of the copulation. These copulation calls are thought to be sexually selected traits that advertise female sexual receptivity to stimulate male–male competition and/or sperm competition—and ultimately obtain higher quality offspring [7–10], or to confuse paternity and reduce infanticide risk [11]. However, for subordinate females, it may not always be advantageous to signal their copulations, and they may even benefit from concealing or restraining their sexual activity in some contexts, to escape aggression from harassing males and dominant females. In line with this, females and subordinate males are more likely to copulate and emit copulation calls when dominant males are out of sight in wild geladas (*Theropithecus gelada*) [12] and in captive macaques (*Macaca mulatta* and *Macaca fascicularis*) [6,13,14], while in chimpanzees (*Pan troglodytes*), the presence of high-ranking females around a mating couple inhibits the emission of copulation calls [15,16].

Further studies are needed to decipher the generality and evolutionary consequences of social influences linked to the presence or absence of third party individuals on female sexual behaviour in promiscuous species. While several reports have shown that dominant males inhibit the sexual behaviour of group members [6,12,14], studies on the effects of dominant female bystanders remain rare (but see [13,15]). Examining in parallel the effect of bystanders from both sexes may thus shed light on the relative importance of the social constraints at play. Moreover, previous studies focusing on social influence on copulations have rarely investigated whether the male or the female mating partner is the most inhibited by bystanders, which would enhance our understanding of the proximate and evolutionary determinants of such phenomena.

This study examines the relative importance of male and female bystanders on female mating behaviour in wild chacma baboons (*Papio ursinus*). Chacma baboons live in multimale–multifemale groups and mate promiscuously. Previous research has shown that adult males direct regular aggression towards oestrous females as a form of sexual intimidation [17], and mate-guard them near ovulation [18]. In addition to this direct coercion—where males are aggressive towards females to increase their own reproductive success—it is possible that males also use indirect coercion, by punishing oestrous females following copulations with rivals to dissuade them from mating with those males again. Social constraints on female sexual behaviour do not arise exclusively from adult males, as oestrous females also face intense aggression from other females, reflecting both mating competition and reproductive suppression over paternal care [19–21]. In particular, pregnant and lactating females are often closely associated with an adult male, usually their offspring's sire [22,23], who provides them and their offspring with paternal services such as protection against predators and conspecifics [24–26]. These non-fertile females harass oestrous females who attempt to mate with the sire of their offspring, which decreases the victim's chances of conception with him [19]. The mechanism mediating this reproductive suppression is not yet elucidated and could include interference in copulations and/or a stress-induced disruption of reproductive physiology.

Female strategies to escape or alleviate those social constraints on their sexual behaviour have not been investigated so far. Despite female–female harassment and male coercion, female baboons utter conspicuous copulation calls at almost every copulation [8], but they may avoid copulating in the presence of harassers. We hypothesize that oestrous females will be less likely to initiate copulations in the presence of male bystanders (hypothesis 1, H1) due to sexual coercion [17] and/or higher-ranking females (H2) due to intrasexual competition over mating opportunities or male social partners [19,20], in order to avoid immediate aggression and copulation interference. We further hypothesize (H3) that male bystanders would inhibit the sexual behaviour of oestrous females more strongly than female bystanders given the importance of sexual dimorphism in this species, where male aggression is the main source of injury for oestrous females [17]. In the course of these analyses, we consider two additional issues. First, that these inhibitory effects will likely be altered by the mate-guarding status of oestrous females, because mate-guarded females may be less able to modulate the occurrence and timing of copulations compared to unguarded females and may further benefit from the protective effect of their mate-guard. We therefore investigate the interaction between mate-guarding status and social influences in our models. Second, that apparent social constraints on sexual behaviour could arise from other aspects affecting all copulations, rather than from active female strategies. For example, if copulations always occur on the periphery of the group, fewer neighbours would be present without involving inhibition. To rule out such a possibility, we further compare social influences on female- and male-initiated copulations: similar social influences on both sexes would raise questions over the interpretation that these effects specifically reflect a female strategy.

## 2. Material and methods

### 2.1. Data collection

Data were collected in June–October 2013 and May–November 2014 from two groups of wild chacma baboons living at Tsaobis Nature Park, Namibia (for details of the site and population, see [27]). Group composition is given in electronic supplementary material, table S1. All individuals were recognizable and habituated to observations at close range. The ages of individuals (in years) were known from long-term life-history records. Males were considered adult at eight years of age and females when they reached menarche [28]. Female dominance ranks were established using *ad libitum* and focal observations of approach-avoid interactions (displacements, supplants) and agonistic interactions (attacks, chases, threats) (see electronic supplementary material, appendix 1).

In baboons, sexually receptive females develop perineal swellings during oestrus, which increase in size as ovulation approaches [29]. Trained observers recorded the swelling size of females daily through a visual assessment [29], using a 7-point scale scoring system. Mate-guarding episodes (or ‘consortships’) were defined as periods when an oestrous female is constantly followed by a male who mates exclusively with her [30]. The formation of consortships, and changes in the identity of partners involved, were monitored *ad libitum* on a daily basis for all females of the group, and mate-guarding associations were further confirmed at the start of each focal observation of an oestrous female to ensure the accuracy of our records.

Observers on foot followed both groups daily from dawn to dusk, conducting focal animal sampling on all adult females and males. All observers (except AB) were blind with respect to the research questions. Focal follows lasted 60 min on average and were spread equally across the day (split into four 3-h time blocks: 6.00–9.00 a.m., 9.00–12.00, 12.00–15.00, 15.00–18.00). Focal individuals were chosen in a semi-random manner, in order to balance observation time equally across individuals and time periods, and each individual was never sampled more than once a day. We collected *N* = 487 observations of 32 oestrous females (mean ± s.d.: 15.2 ± 8.8 per individual) and *N* = 551 observations of 25 adult males (mean ± s.d.: 22.0 ± 9.3 per individual). All occurrences of copulations were recorded (with both juvenile and adult males), together with the identity of sexual partners and who was responsible for initiating the copulation. Copulations were considered initiated by females when they approached first and presented their hindquarters to males or when they solicited copulations using facial expressions (come-here faces and/or lip-smacks), and by males when they approached first and grabbed the female's hindquarters or used the same facial expressions. Unclear cases were recorded as ‘unknown initiator’. Immediately after each copulation (i.e. within 30 s maximum), we performed a comprehensive audience scan (copulation scan) recording the identity of all adult males and females present within 0–5 m of the mating couple during the copulation. Audience scans were also performed routinely every 10 min (non-copulation scans). In both copulation and non-copulation scans, individual neighbours were recorded even if they were out of sight of the focal subject (e.g. because of vegetation). Non-copulation scans occurring within less than 10 min of copulations were removed to ensure that non-copulation scans referred only to non-mating contexts. For mate-guarded females, the male consort was omitted from the ‘non-copulation scans' in consistency with ‘copulation scans’ (where the copulating consort is not included) to avoid an artificial increase of the mean number of adult males around mate-guarded females in non-copulation scans. Table 1 summarizes the number of copulation and non-copulation scans where at least one adult male and higher-ranking female were present within 5 m of the focal female.

**Table 1.** Audience composition of non-copulation scans, female-initiated copulation scans and male-initiated copulation scans.

	non-copulation	female-initiated copulations	male-initiated copulations
total no. of scans^a^	2076	173	290
no. of scans with at least one higher-ranking female bystander^a^	254	17	35
average no. of higher-ranking female bystanders^b^	0.16 ± 0.20	0.11 ± 0.16	0.08 ± 0.13
average no. of higher-ranking female bystanders for unguarded females^b^	0.21 ± 0.31	0.06 ± 0.15	0.08 ± 0.21
average no. of higher-ranking female bystanders for mate-guarded females^b^	0.13 ± 0.18	0.18 ± 0.25	0.13 ± 0.24
no. of scans with at least one male bystander^a^	378	3	24
average no. of male bystanders^b^	0.19 ± 0.11	0.01 ± 0.03	0.09 ± 0.22
average no. of male bystanders for unguarded females^b^	0.33 ± 0.14	0.01 ± 0.04	0.05 ± 0.15
average no. of male bystanders for mate-guarded females^b^	0.02 ± 0.03	0.00 ± 0.01	0.08 ± 0.21

^a^These figures are calculated as the sum of available scans for each category of scans in the models.

^b^These figures are calculated by averaging the number of male and female bystanders for each focal female and for each type of scan (non-copulation scans, *N* = 32 focal females; female-initiated scans, *N* = 24 focal females; and male-initiated scans, *N* = 24 focal females). We provide the mean value ± standard deviation.

### 2.2. Statistical analyses

We tested whether male and female bystanders inhibit the copulations of oestrous females by comparing the composition of the audience (i.e. individuals standing in the immediate proximity of the mating couple, and therefore presumably in visual and hearing range of their behaviour) in copulation versus non-copulation scans. A binomial Generalized Linear Mixed Model (GLMM) was run with a logit link function, using the occurrence of female-initiated copulations as the response variable (1/0, copulation present/absent, drawing on female-initiated copulation scans and female-focal non-copulation scans, respectively). We included female-initiated copulations collected during both male and female observations (because the audience during copulation is the same for the male and female mating) but used only non-copulation scans from female observations, to control for the regular audience of oestrous females only. Fixed factors included:

— the number of adult male bystanders
— the number of female bystanders that outranked the focal female
— the mate-guarding status of the focal female (0: unguarded; 1: mate-guarded). Unguarded females could copulate with any juvenile or adult male. Mate-guarded females could only copulate with their consort male (we had only 2 cases of extra pair copulations out of 397 copulations; these two cases were removed from subsequent analyses).
— we also tested the significance of the interactions between the mate-guarding status of the focal female and the number of (1) male bystanders and (2) higher-ranking female bystanders to test if social influences vary according to mate-guarding status.
— the swelling size of the focal female (to control for increasing sexual activity as females approach ovulation)
— the relative rank and age of the focal female (to control for the fact that sexual activity may differ among females of various ranks and ages)
— the group identity and year of study (to control for other sources of variation in the rates of copulation across groups and years).

Random factors comprised the identity of the focal female, the identity of the focal observation and the date of observation.

To ensure that any observed social influence on the probability of copulation reflected the responses of oestrous females to their audience rather than any general property of copulations, we further compared social influences on female- and male-initiated copulations to the same non-copulation scans (in this case the regular audience of oestrous females). We ran a control model (same fixed and random effects) with male-initiated copulations as the response variable (1/0, copulation present/absent, drawing on male-initiated copulation scans and female-focal non-copulation scans respectively) and fitted the same fixed and random effects.

All GLMMs were run using the glmer function of the lme4 package [31] in R v. 3.4.1 [32]. All quantitative variables were *z*-transformed to have a mean of zero and a standard deviation of one (by subtracting the mean from each value and dividing by the standard deviation) to facilitate model convergence. The significance of the fixed factors was tested using a likelihood ratio test, ‘LRT’ (assuming an asymptotic chi-square distribution of the test statistic), and using the full model to avoid problems arising from stepwise model-selection procedures [33,34]. We only tested two-way interactions for which we had a clear prediction. Non-significant interactions were omitted from the full model to limit risks of over-parametrization and facilitate the interpretation of simple effects. The significance of the fixed factors was assessed by computing their 95% Wald confidence intervals (using the confint.merMod function) and by checking that they did not cross zero. To diagnose the presence of multicollinearity, we calculated the variance inflation factor for each predictor. The maximal value of VIF was 1.85 for the female-initiated model and 1.73 for the male-initiated model, which are well below 3, and thus do not indicate serious multicollinearity [35]. The correlation coefficient between the number of male and higher-ranking female bystanders among audience scans was 0.22 for both the female-initiated and male-initiated models, which falls below the critical threshold of 0.70 [36].

## 3. Results

**Figure 1.**
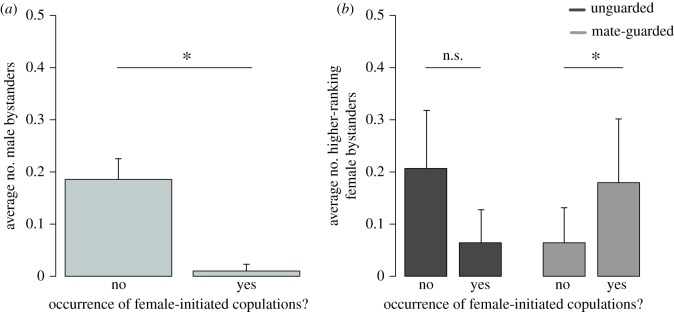
Barplot of the mean number of (*a*) male bystanders and (*b*) higher-ranking female bystanders during non-copulation and female-initiated copulation scans. Barplots are drawn from the raw individual means. Error bars indicate the standard error of the mean. ‘*’: *p* < 0.05, ‘n.s.’: *p* > 0.05.

Oestrous females initiated fewer copulations when the audience contained more adult males (table 2 and figure 1*a*), and this was true for both unguarded and mate-guarded females (comparison of models with and without an interaction between the number of male bystanders and mate-guarding status: $\chi_{1}^{2}=2.06,$ *p* = 0.151). By contrast, the effect of higher-ranking female bystanders differed markedly with the mate-guarding status of oestrous females (comparison of models with and without an interaction between the number of female bystanders and mate-guarding status: $\chi_{1}^{2}=7.64,$ *p* = 0.006 and table 2). We ran the GLMM separately for unguarded and mate-guarded females to explore these differences. Mate-guarded females initiated more copulations when the audience contained more higher-ranking females (electronic supplementary material, table S2; figure 1*b*). By contrast, unguarded females rarely initiated copulations in the presence of dominant females (mean number of higher-ranking females during female-initiated copulations: 0.06 ± 0.15 compared to 0.21 ± 0.31 outside mating context, table 1 and figure 1*b*), though this trend did not reach statistical significance (see electronic supplementary material, table S2). The contrasting mating patterns observed between mate-guarded and unguarded females did not arise from passive differences in their audiences in non-copulation scans because mate-guarding females have a similar number of higher-ranking females in proximity during non-copulation scans (mean number ± s.d.: 0.13 ± 0.18, table 1) than unguarded females (0.21 ± 0.31, one sample *t*-test, *t* = 1.17, d.f. = 48.70, *p* = 0.246).

**Table 2.** Social influences on the occurrence of female-initiated copulations. Tests are based on 24 females, 43 males copulating and 173 copulation scans (70 of which occurred with juveniles), including 73 copulations involving mate-guarded females and 100 involving unguarded females, and 2076 non-copulation scans. Significant variables appear in bold. s.e.: standard error; LRT: statistic of a likelihood ratio test, d.f.: degrees of freedom. LRT, d.f. and *p*-values are not given for fixed effects involved in a significant interaction.

response variable	fixed factor	estimate	s.e.	95% confidence interval	LRT	d.f.	*p*-value
occurrence of female-initiated copulations (0/1)	number of higher-ranking female bystanders	−0.34	0.36	[−1.05 ; 0.37]			
	number of male bystanders	−2.32	0.44	**[−3.19; −1.45]**	49.02	1	**<0**.**001**
	female is mate-guarded^a^	−0.85	0.30	**[−1.44; −0.27]**			
	number of higher-ranking female bystanders : mate-guarding^a^	1.08	0.43	**[0.24; 1.92]**	7.64	1	**0**.**006**
	swelling size	0.45	0.17	**[0.11; 0.79]**	7.73	1	**0**.**005**
	relative rank	−0.56	0.47	[−1.49; 0.37]	1.75	1	0.186
	age	−0.68	0.49	[−1.64; 0.28]	2.29	1	0.130
	troop^b^	0.05	0.46	[−0.85; 0.94]	0.00	1	0.950
	year^c^	2.55	0.66	**[1.26; 3.84]**	39.22	1	**<0**.**001**

^a^Reference category: female is unguarded.

^b^Reference category: J troop.

^c^Reference category: 2013.

**Figure 2.**
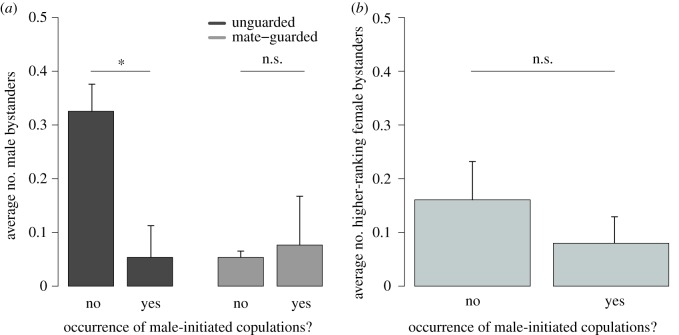
Barplot of the mean number of (*a*) male bystanders and (*b*) higher-ranking female bystanders during non-copulation and male-initiated copulation scans. Barplots are drawn from the raw individual means. Error bars indicate the standard error of the mean. ‘*’: *p* < 0.05, ‘n.s.’: *p* > 0.05.

For male-initiated copulations, the effect of male bystanders differed according to the mate-guarding status of the female (comparison of models with and without an interaction between the number of male bystanders and mate-guarding status: $\chi_{1}^{2}=15.59,$ *p* < 0.001 and table 3): males were less likely to initiate copulations with unguarded females in the presence of male bystanders, while mate-guarding males were not inhibited by male bystanders (table 3 and figure 2*a*; electronic supplementary material, table S3). Female bystanders did not influence male-initiated copulations with guarded or unguarded females (comparison of models with and without an interaction between the number of female bystanders and mate-guarding status: $\chi_{1}^{2}=1.60,$ *p* = 0.205, figure 2*b*), suggesting that the reported social influences on copulations are not simply a general property of copulations. In addition, female-initiated and male-initiated copulations did not differ in terms of the female audience (0.11 versus 0.08 female bystanders, table 1, one sample *t*-test, *t* = 0.69, d.f. = 45.14, *p* = 0.491), but female-initiated copulations have substantially fewer male bystanders than male-initiated copulations (0.01 versus 0.09 male bystanders, table 1, one sample *t*-test, *t* = −2.04, d.f. = 30.54, *p* = 0.050), suggesting that the effect of a male audience on female-initiated copulations reflects an active female avoidance strategy.

**Table 3.** Social influences on the occurrence of male-initiated copulations. Parameters and tests are based on 30 focal females, 51 males copulating and 290 copulation scans (124 of which occurred with juveniles), including 151 copulations involving mate-guarded females and 139 involving unguarded females, and 2076 baseline scans. Significant variables appear in bold. s.e.: standard error, LRT: statistic of a likelihood ratio test, d.f.: degrees of freedom. LRT, d.f. and *p*-values are not given for fixed effects involved in a significant interaction.

response variable	fixed factor	estimate	s.e.	95% confidence interval	LRT	d.f.	*p*-value
occurrence of male-initiated copulations (0/1)	number of higher-ranking female bystanders	0.09	0.16	[−0.23; 0.41]	0.30	1	0.582
	number of male bystanders	−1.17	0.25	**[−1.67; −0.68]**			
	female is mate-guarded^a^	0.29	0.26	[−0.22 ; 0.79]			
	number of male bystanders: mate-guarding^a^	1.78	0.45	**[0.90; 2.66]**	15.59	1	**<0**.**001**
	swelling size	0.58	0.25	**[0.09; 1.07]**	6.41	1	**0**.**011**
	relative rank	−0.77	0.45	[−1.64; 0.11]	3.55	1	0.059
	age	−0.70	0.47	[−1.61; 0.22]	2.77	1	0.096
	troop^b^	−0.34	0.43	[−1.18; 0.50]	0.87	1	0.350
	year^c^	1.51	0.35	**[0.83; 2.20]**	24.92	1	**<0**.**001**

^a^Reference category: female is unguarded.

^b^Reference category: J troop.

^c^Reference category: 2013.

## 4. Discussion

Our results show that oestrous females modulate their mating activity in the presence of adult male bystanders in wild chacma baboons. Although male mating interference and punishment after copulation with rival males are rare in baboons [17], males that repeatedly harass oestrous females before ovulation increase their mating success with the victim during the ovulatory period, in a form of direct sexual coercion [17]. In this context, our current results suggest that females decrease sexual solicitations in front of male bystanders to avoid subsequent aggression (even if that aggression does not immediately follow a copulation), as well as the associated risk of injuries [17]. This suggests an additional, indirect form of sexual coercion in baboons, such that repeated male aggression does not only encourage oestrous females to mate with them (intimidation) but further dissuades them from mating with their rivals (punishment). Although we did not find evidence of immediate punishment (i.e. within 15 min) following copulations with rival males in a previous study [17], we cannot discard the possibility that some form of punishment exists, which may be non-systematic and delayed. Even if the risk of punishment is substantial, our ability to detect it is probably hampered by the evolution of effective counter-strategies such as female avoidance of potential harassers, as suggested by these results. We further found that male-initiated copulations with unguarded females (mainly by juveniles) are also inhibited by the presence of adult males. This suggests that the suppressive effect of male bystanders extends to most group members except high-ranking adult males and that most copulations may occur away from them. Indeed, only copulations initiated by mate-guarding males, who are typically high-ranking, were unaffected by the male audience. Overall, our results suggest that the effects of male bystanders on mating activity are stronger than those of female bystanders (based on the relative value of the estimate of the effect of male and female bystanders in table 2), possibly reflecting the substantial sexual dimorphism in body and canine size; males can induce severe and life-threatening injuries to females. Such inhibiting effects of dominant males have been found in many polygynous mammals (e.g. gelada: [12], rhesus macaque: [6,14]; long-tailed macaque: [6,13]; Japanese macaque, *Macaca fuscata*: [37], Southern elephant seal, *Mirounga leonina:* [38]) and may represent an important underpinning mechanism in the male reproductive skew observed in these species.

Despite previous findings of harassment from dominant females toward subordinate oestrous females in chacma baboons [19–21], we found no evidence that oestrous females inhibit their sexual behaviour in the presence of dominant female bystanders. Instead, it seems that oestrous females, and particularly unguarded females, avoid the proximity of high-ranking females both in copulation and non-copulation contexts, suggesting that high-ranking females harass them continuously, and not just after a copulation event. Our previous study revealed that pregnant and lactating females forming a close bond with an adult male harass oestrous females who attempt to mate with him, thereby decreasing their chances of conception [19]. Together with these new results, this suggests that the main proximate mechanism mediating female-induced reproductive suppression is not copulation interference. Rather, chronic stress resulting from repeated harassment directed towards oestrous females across their cycle (and not just after copulations) may downregulate the reproductive physiology of the victims [39].

Surprisingly, we found that mate-guarded females initiated more copulations in the presence than in the absence of higher-ranking females. There was no such effect of high-ranking females on male-initiated copulations in a context of mate-guarding, suggesting that it is not a passive consequence of changes in a female's social environment associated with mate-guarding episodes. This intriguing result suggests that the mating activity of oestrous females is enhanced by the proximity of high-ranking rivals in a mate-guarding episode: the presence of rivals may stimulate sexual activity in a context of reproductive competition where oestrous females feel protected by the proximity of their male consort. Alternatively, oestrous females may solicit more copulations with their consort when they feel threatened by the proximity of high-ranking females in an attempt to keep him close, under a bodyguard scenario. Further analyses on larger sample sizes, that take into account the intensity of the competition between females by examining social influences on sexual activity in relation to the friendship status of the female bystanders (i.e. testing whether this effect is stronger when female bystanders are friends with the mating male, and therefore more likely to direct aggression towards the female) could help to elucidate this possibility.

The proximate mechanisms underlying the social inhibition of mating activity may result from simple processes, where females avoid copulating in the presence of certain bystanders or take advantage of their absence. For example, subordinate male baboons monitor temporary separations between a dominant male and his mate-guarded female [40], possibly to identify opportunities for copulations and/or sneaky matings. The frequency of agonistic interactions faced by oestrous females may similarly force them to monitor constantly the presence of conspecifics in close proximity. Alternatively, this social inhibition may result from cognitively complex strategies, such as tactical deception where copulation partners increase their spatial distance [6] or hide intentionally [5,12] from specific bystanders. Further research will help to clarify the mechanisms at play in baboons, but this study indicates that passive social influences on female mating strategies may be effective at shaping female sexual behaviour, including the frequency of matings and the identity of mating partners, in promiscuous societies.

## 5. Conclusion

Despite displaying conspicuous copulations, female chacma baboons seem to restrain their sexual activity in the presence of male bystanders. Why do females hide from adult bystanders if they signal their copulations so loudly? This apparent paradox may arise from a trade-off between the long-range audience targeted by their copulatory signal and their immediate social environment. Copulatory signals are thought to attract preferred mating partners—often high-ranking males—by signalling female fertility (e.g. [10,41]). However, in the context of a coercive society, it may be safer for females to avoid copulating in the immediate surroundings of high-ranking males if they do not mate with them. Male recurrent aggression towards oestrous females may not only encourage females to mate with them but further discourage them to mate with rivals. In promiscuous societies, females often seem to have little sexual freedom and may need to use complex tactics to implement their strategies in an equally complex social landscape.

## Supplementary Material

Audience effect on mating behaviour_sub 3_Supplementary.docx

## Data accessibility

Data are available through Dryad Digital Repository (https://github.com/AliceBaniel/Social-influences-on-baboon-matings).
