# Supplementary material for: Oestrous females avoid mating in front of adult male bystanders in wild chacma baboons
Source: R Soc Open Sci. 2019 Jan 16;6(1):181009. doi: 10.1098/rsos.181009 (PMC6366197; doi:10.1098/rsos.181009)
Supplement: Audience effect on mating behaviour_sub 3_Supplementary.docx [file rsos181009supp1.docx]

**Supplementary material**

for

**Oestrous females avoid mating in front of adult male bystanders in wild chacma baboons**

**Alice Baniel^§^, Axelle Delaunay, Guy Cowlishaw, Elise Huchard**

**^§^contact: alice.baniel@gmail.com**

**This file includes:**

**Table S1: Demography of J and L groups in the study periods (page 2).**

**Table S2. Effect of male and higher-ranking female bystanders on the occurrence of female-initiated copulations, for unguarded and mate-guarded females separately (page 3).**

**Table S3. Effect of male and higher-ranking female bystanders on the occurrence of male-initiated copulations, for unguarded and mate-guarded females separately (page 4).**

**Appendix 1. Establishment of female dominance ranks (page 5)**

**Table S1:** Demography of J and L groups in 2013 and 2014. Demography varies due to emigrations, immigrations, births, deaths, and maturations.

| Year | Number of adult males | | Number of adult females | | Number of juveniles* | | Total group size | |
| --- | --- | --- | --- | --- | --- | --- | --- | --- |
|  | J group | L group | J group | L group | J group | L group | J group | L group |
| 2013 | 7-10 | 9-11 | 17 | 18-19 | 29-32 | 31-33 | 53-59 | 58-63 |
| 2014 | 7-8 | 9 | 18 | 17-19 | 35 | 29 | 60-61 | 55-57 |

*Subadult males (i.e. between 4 and 8 years old) are counted as juveniles in this study.

**Table S2. Effect of male and higher-ranking female bystanders on the occurrence of female-initiated copulations, for unguarded and mate-guarded females separately.** Parameters and tests are based on 22/17 focal females, 38/15 males copulating, 100/73 copulation scans, 1074/1002 baseline scans for unguarded/mate-guarded females respectively. Significant variables appear in bold. SE: Standard Error, LRT: statistic of a likelihood ratio test, df: degrees of freedom.

|  |  | Unguarded females | | | | | |  | Mate-guarded females | | | | | |
| --- | --- | --- | --- | --- | --- | --- | --- | --- | --- | --- | --- | --- | --- | --- |
| Response variable | Fixed factor | Estimate | SE | 95% confidence interval | LRT | df | P-value |  | Estimate | SE | 95% confidence interval | LRT | df | P-value |
| Occurrence of female-initiated copulation (0/1) | No of higher-ranking female bystanders | -0.27 | 0.38 | [-1.01 ; 0.46] | 0.54 | 1 | 0.464 |  | 0.68 | 0.25 | **[0.18 ; 1.17]** | 7.86 | 1 | **0.005** |
|  | No of male bystanders | -2.52 | 0.51 | **[-3.51 ; -1.52]** | 47.97 | 1 | **<0.001** |  | -1.47 | 1.10 | [-3.63 ; 0.70] | 2.23 | 1 | 0.135 |
|  | Swelling size | 0.70 | 0.23 | **[0.25 ; 1.15]** | 11.10 | 1 | **0.001** |  | 0.25 | 0.32 | [-0.37 ; 0.87] | 0.70 | 1 | 0.403 |
|  | Relative rank | -0.34 | 0.53 | [-1.37 ; 0.69] | 0.54 | 1 | 0.462 |  | -0.66 | 0.57 | [-1.77 ; 0.46] | 1.58 | 1 | 0.208 |
|  | Age | -0.30 | 0.54 | [-1.37 ; 0.77] | 0.37 | 1 | 0.544 |  | -0.96 | 0.60 | [-2.14 ; 0.23] | 3.08 | 1 | 0.079 |
|  | Troop^a^ | -0.13 | 0.51 | [-1.12 ; 0.87] | 0.10 | 1 | 0.747 |  | 0.48 | 0.55 | [-0.61 ; 1.56] | 0.90 | 1 | 0.343 |
|  | Year^b^ | 2.17 | 0.75 | **[0.71 ; 3.64]** | 18.19 | 1 | **<0.001** |  | 2.50 | 0.75 | **[1.02 ; 3.98]** | 18.30 | 1 | **<0.001** |

^a^ Reference category: J troop

^b^ Reference category: 2013

**Table S3. Effect of male and higher-ranking female bystanders on the occurrence of male-initiated copulations, for unguarded and mate-guarded females separately.** Parameters and tests are based on 31/28 focal females, 45/15 males copulating, 139/151 copulation scans, 1074/1002 baseline scans for unguarded/mate-guarded females respectively. Significant variables appear in bold. SE: Standard Error, LRT: statistic of a likelihood ratio test, df: degrees of freedom.

|  |  | Unguarded female | | | | | |  | Mate-guarded female | | | | | |
| --- | --- | --- | --- | --- | --- | --- | --- | --- | --- | --- | --- | --- | --- | --- |
| Response variable | Fixed factor | Estimate | SE | 95% confidence interval | LRT | df | P-value |  | Estimate | SE | 95% confidence interval | LRT | df | P-value |
| Occurrence of male-initiated copulation (0/1) | Number of higher-ranking female bystanders | 0.00 | 0.22 | [-0.43 ; 0.44] | 0.00 | 1 | 1.000 |  | 0.21 | 0.23 | [-0.24 ; 0.66] | 0.94 | 1 | 0.333 |
|  | Number of male bystanders | -1.14 | 0.23 | **[-1.60 ; -0.68]** | 27.90 | 1 | **<0.001** |  | 0.56 | 0.39 | [-0.20 ; 1.32] | 2.23 | 1 | 0.135 |
|  | Swelling size | 0.40 | 0.20 | **[0.01 ; 0.79]** | 4.43 | 1 | **0.035** |  | 0.40 | 0.26 | [-0.11 ; 0.91] | 2.98 | 1 | 0.084 |
|  | Relative rank | -0.50 | 0.51 | [-1.51 ; 0.50] | 1.23 | 1 | 0.267 |  | -0.71 | 0.52 | [-1.74 ; 0.31] | 2.25 | 1 | 0.134 |
|  | Age | -0.90 | 0.53 | [-1.94 ; 0.14] | 3.63 | 1 | 0.057 |  | -0.40 | 0.54 | [-1.46 ; 0.67] | 0.62 | 1 | 0.432 |
|  | Troop^a^ | -0.46 | 0.48 | [-1.41 ; 0.48] | 1.19 | 1 | 0.275 |  | 0.10 | 0.51 | [-0.89 ; 1.10] | 0.04 | 1 | 0.836 |
|  | Year^b^ | 0.68 | 0.52 | [-0.33 ; 1.70] | 2.61 | 1 | 0.106 |  | 1.96 | 0.48 | **[1.02 ; 2.90]** | 21.86 | 1 | **<0.001** |

^a^ Reference category: J troop

^b^ Reference category: 2013

**Appendix 1. Establishment of female dominance ranks**

Female dominance ranks were established using both *ad libitum* and focal observations of dyadic approach-avoid interactions (*supplants*, when one animal actively displaces another to take its place; *displacements*, when one animal passes close to another and makes it move away) and dyadic agonistic interactions (*attacks*, any agonistic physical contact including hits, bites, or grabbing movements; *chases*, when one animal chases another for a distance of at least 3m; *threats*, including staring, head bobbing, and ground sweeping while oriented toward the targeted individual). We calculated female dominance hierarchies separately and in each year using Matman 1.1.4 (Noldus Information Technology 2003). We calculated and tested the adjusted linearity index h, corrected for unknown relationships. Hierarchies were always linear (*N*_2013_ = 367 interactions, *N*_2014_ = 1259 in group L; *N*_2013_ = 590, *N*_2014_ = 978 in group J, Landau’s linearity index h: *p* < 0.05 in all cases). In the following analyses, we used relative rank to control for variation in group size. To calculate relative rank, absolute ranks were standardised to vary between 0-1, using the formula: 1-((1-*r*)/(1-*n*)), where *r* is the absolute rank of an individual and *n* is the total number female in the group.
